# Future Land Use for Insect Meat Production Among Countries: A Global Classification

**DOI:** 10.3389/fnut.2021.661056

**Published:** 2021-05-25

**Authors:** Hideyuki Doi, Randy Nathaniel Mulia

**Affiliations:** Doi Laboratory, Graduate School of Simulation Studies, University of Hyogo, Kobe, Japan

**Keywords:** insect, livestock, climate change, food security, global analysis, land area, insect meat, entomophagy

## Abstract

A potentially suitable alternative to reduce land use by livestock production is insect meat production. However, land use predictions for insect meat production, which are important in the planning of food production strategies in each country, have not been well-performed. To consider the strategy of insect meat production with regard to land use, the categorical perspectives of countries would be highly useful. Here, using previous simulation results, we used random forest machine learning to classify the potential land use of 157 countries for insect meat production under future climate change. From the categorical maps, we showed the global distribution of five different country categories and found that the land area of the countries may be an important factor in considering the future increase in insect meat production. Our classification could be used to help formulate future food policies by considering the increase in insect meat production in each country, as well as regionally and/or globally.

## Introduction

Livestock provide almost 25% of all dietary protein and 15% of dietary energy; consequently, livestock production has significant environmental impacts (1, 2). Despite improvements in crop yields and livestock production efficiency, the global cropland area is expanding, increasing by 464 Mha between 1961 and 2011 (3), with almost a third of the harvested crops used for livestock feed (1). Therefore, increasing of livestock production would induce land use, including cropland area, and impact on the sustainable land use (1).

In recent decades, land use change has accounted for 10–12% of total anthropogenic CO_2_ emissions (4). A combination of land use change and other emissions has increased the percentage of agriculture-related global anthropogenic greenhouse gas (GHG) emissions to between 17 and 32% (4). Thus, the increase in demand for agricultural products, especially livestock products (e.g., meat, milk, and eggs), could significantly alter GHG emissions (5). Furthermore, increasing the availability of agricultural land provides options for climate change mitigation measures such as afforestation and bioenergy (6).

Entomophagy has long been considered as an alternative or additional source of animal protein. More than 1,900 species of insects have been reported to be used for food, and insects constitute a part of the traditional diet of at least two billion people (7). Despite historical references to the use of insects as food, it is only recently that entomophagy has begun to receive global attention and emerged as a new trend in food science (1, 2, 8–10). Numerous edible insects have traditionally been collected from forest habitats, but innovations in systems for the mass rearing of insect larvae have been introduced and are already in use (11, 12). Insect farms are currently in the development stage and are beginning to create a completely unique agricultural sector. Entomophagy potentially provides redundancy and diversity in the food system, along with higher production rates (13–15).

The environmental benefits of rearing insects for food and feed are attributed to the high feed conversion efficiency of insects. Crickets, for example, require only 1.7 kg of feed per kilogram of body weight (16). Insects have been reported to produce fewer GHG emissions and less ammonia than cattle and pigs, which can significantly reduce the land and water use of livestock production (13, 14). In addition, insects can be reared in organic side streams (including human and animal waste), which can potentially increase profitability (17). Thus, entomophagy positively contributes to the sustainability of human society, land use, and the environment. In particular, given the COVID-19 pandemic in 2020, entomophagy potentially has several merits for food security and human society, considering its environmental benefits and minimal risk of zoonotic disease transmission (10).

The environmental benefits of entomophagy are attributed to its higher feed conversion efficiency and lower land use requirements for the production weight, protein, and energy than conventional livestock (7). Therefore, entomophagy contributes positively to the sustainability of human society and land use (13, 15). To reduce land use by livestock production, insect meat production is potentially a suitable alternative. However, land use predictions for insect meat production, which are important in the planning of food production strategies in each country, have not been well-performed. Mulia and Doi (15) examined the land use effectiveness of insects as food in combination with the use of other conventional meats under different climate change scenarios [Special Report on Emissions Scenarios (SRES)]. This study was an initial step to considering the land use for insect production; however, it only showed the land use simulation of each country in 2100.

To consider land use strategies for insect meat production, the categorical perspectives among countries would be useful for policymaking and future studies. In this study, we focused on the global category of land use for insect meat production under different climate change scenarios. Here, using the results of our random forest machine learning simulation, we performed a new analysis and classification among countries with regard to land use for insect meat production and consider future strategies for insect meat production. We classified the countries in globe for discussing the land use issue on future livestock production with increasing of insect meat production ratio.

## Summary of Land Use Simulation

In this study, we used the simulation results from Mulia and Doi (15). In brief, they developed a simulation model using the calorie demand model by Bodirsky et al. (18) to calculate future world calorie demand and livestock percentage. The model assumes that the total calorie demand increases with the economic growth of the countries. For low-income countries, the proportion of animal-based calories is estimated to rise with income, while for high-income countries, it is expected to decrease with an increase in income due to high-income groups becoming more health-conscious (19). In the simulation, all combinations of the livestock production ratios (beef, pork, lamb, poultry, fish, and insects) were simulated. Then, the land use requirement for total energy demand and livestock share data from the calorie demand model was used to calculate the livestock energy demand globally or of a given area or country. According to Eitelberg et al. (20), the current global cropland area is 1,550 Mha and future expansion may range from 1,552 to 5,131 Mha; the current value was used for the simulation as the assumed global land use limit. The simulation evaluated the land use demand for future livestock production in each country (157 countries) from 2010 to 2100. Here, we used the simulation data of land use demand to categorize the countries.

In accordance with Mulia and Doi (15), we used two scenarios of livestock production ratios: (1) current livestock ratio with almost 0% insect meat production and (2) an increase in insect meat to 10% of total caloric demand as the typical pattern of the simulation by Mulia and Doi (15). The livestock productions in the simulation were beef, pork, lamb, poultry, fish, as well as insects. We simulated land use for production of enough livestock meat to sustain the human society as per the predicted energy demand in four different SRES scenarios from 2020 to 2100. The simulation was performed for each SRES scenario with A1 (economic, global issue), A2 (economic, regional issue), B1 (environment, global issue), and B2 (environment, regional issue) (Table 1); thus, we used the combined land use predictions of A1, A2, B1, and B2 for the following classifications. We analyzed the future land use in 2100 in each country.

**Table 1:** General assumptions of Special Report on Emissions Scenarios (SRES).

	**Economic**	**Environmental**
Globalized	**A1** Rapid economic growth. Population peaks mid-century and declines thereafter. Global convergence, as well as increased cultural and social interactions.	**B1** A rapid change toward the service and information economy and clean technology. Population peaks mid-century and declines thereafter. Global convergence, as well as increased cultural and social interactions.
Regionalized	**A2** Regionally oriented economic development. Continuously increasing population. Self-reliance and preservation of local identities.	**B2** Less rapid and diverse technological change than B1 and A1. Continuously increasing populations. A lower rate of increase than in A2. Locally and regionally oriented environmental protection and social equity.

## Classification Methods

All statistical and graphical analyses were performed using R ver. 4.0.3 (2020) (21). We employed random forest to classify the countries using the above simulation data in the *randomForest* function in “randomForest” ver. 4.6.14 package. We preliminarily performed several models with different numbers of trees, but did not find significant differences, and determined the most appropriate number of trees to be 1,000. Using the random forest classification, we determined the cutting categories using Ward's method regarding the *hclust* function in R. We created the graphics using “ggplot2” ver. 3.3.3. We used map data from “maps” ver. 3.3.0 free license package.

## Classification Results and Discussion

**Figure 1:**
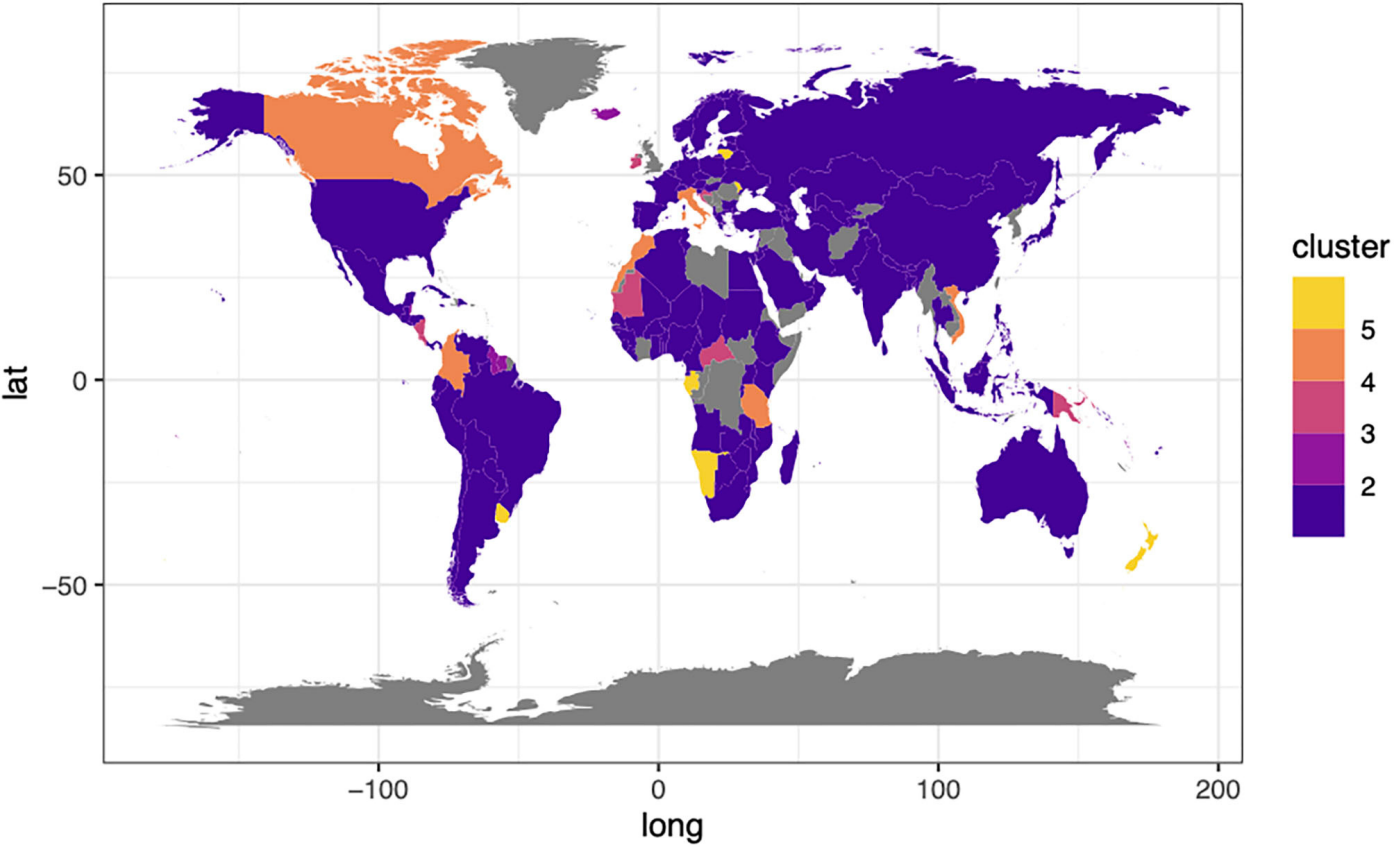
Global map for random-forest classification of land use demand with (1) the current livestock ratio in 2070–2100. Categories 1–5 represent the classification groups with generally increasing land use demand under the various Special Report on Emissions Scenarios (SRES).

**Figure 2:**
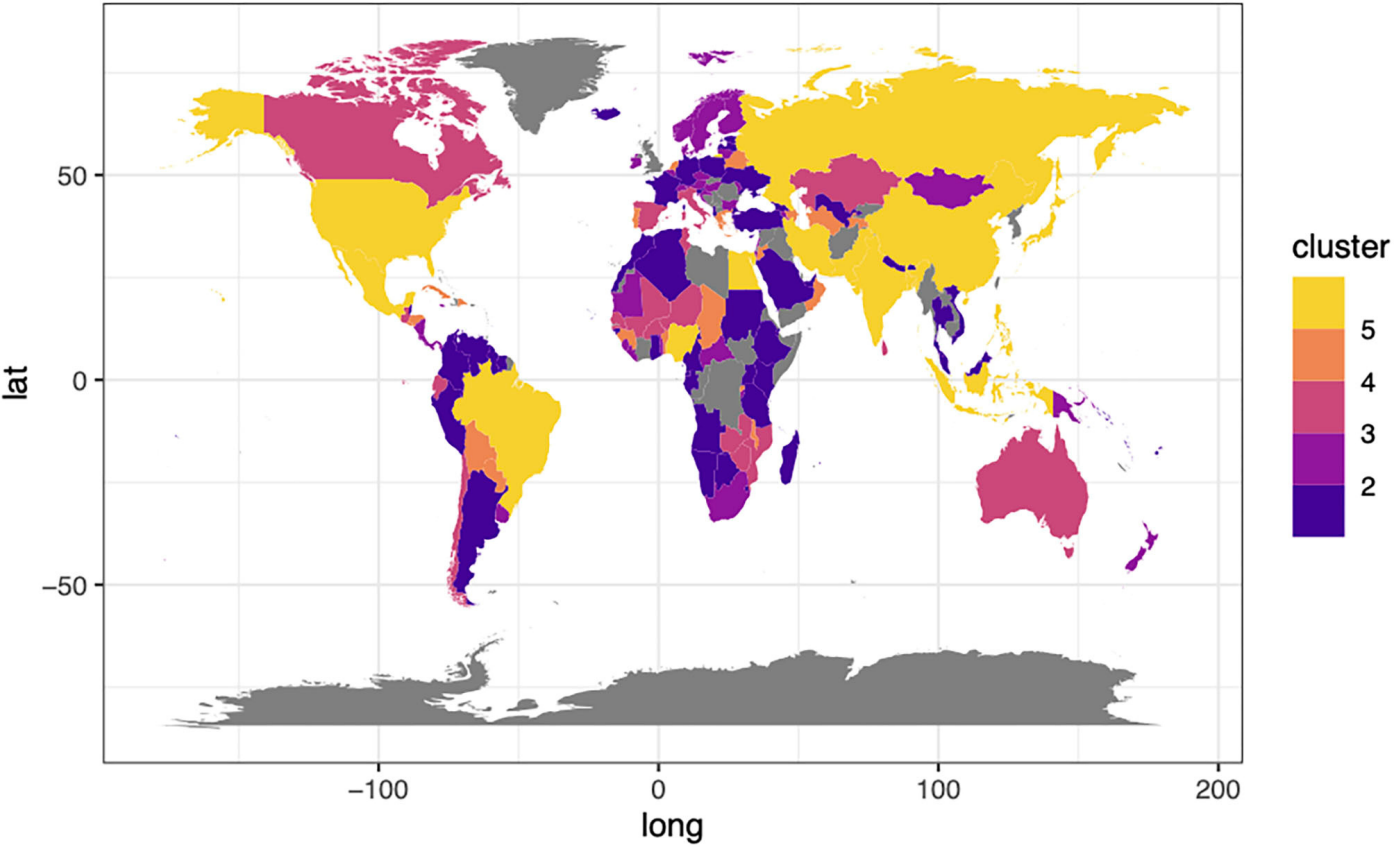
The global map for random-forest classification by land use demand with (2) increased insect meat to 10% in 2070–2100. Categories 1–5 represent the classification groups with generally increasing land use demand under the various Special Report on Emissions Scenarios (SRES).

Our classifications showed a globally variable pattern in the category (Figures 1, 2). In the figures, we should note the land use demand for livestock with increasing category numbers. For the current livestock ratio scenario (scenario 1, almost 0% insect meat production), countries were mainly placed in the same category, including developed and developing countries (Figure 1). This probably means that the current livestock ratio is distributed without regarding the economy and locations, especially in category 1 (Figure 1).

For the scenario in which insect meat production was increased to 10% (scenario 2, increase in insect meat to 10% of total caloric demand), many large-area countries, such as Russia, China, the USA, and India, as well as the continents Europe and Africa, were also included in category 5 (Figure 2). The categories can be considered to represent the importance of increasing insect meat production among countries. In large-area countries, we expected that it may not be necessary to increase insect meat production because the land area is not limited, from the simulation, while in small countries, increasing insect meat production is necessary to consider the land use proportion in each country. Therefore, we expected that, especially in small-area countries, increasing insect meat production may play an important role in land use issues under future climate change scenarios.

In summary, from the categorical maps, we found that the land area of the countries may be an important factor in considering the increase in insect meat production in the future. Land use is a critical issue in food security (1, 3, 22) as well as environmental issues (13, 14, 22). Therefore, mapping with a clear classification would be useful for future studies and policymaking. In this study, we performed the random forest machine learning technique to categorize the future predictions of 157 countries, which could be applied to create categorical maps of other food security and economic issues.

## Conclusion Remarks

Our classification can be used as a reference for future food policies at the country, regional, and/or global levels. We believe that these maps can be used to determine whether sufficient food supplies can be obtained from available livestock land or from insect production. However, it may be difficult to predict future livestock energy demands and the complex structures of food supply and policymaking. In addition, for policymaking, we should consider the demerit of insect production, such as invasive species escaping from insect-rearing factories (23). In the present study, we only focused on the country-level phenomena; however, future studies are required on certain localized factors for each region, such as ecosystem protection and conservation with different priorities and conditions, to develop local scale mapping of the land use demand for future livestock by considering the potential increase in insect production.

## Data Availability Statement

Publicly available datasets were analyzed in this study. This data can be found here: All data used in this study can be downloaded from the data sources, including the websites for the World Bank (https://data.worldbank.org) and Mulia and Doi (15).

## Author Contributions

HD and RM conceived the idea and hypothesis, designed the experiments, and wrote the manuscript. HD analyzed the data. Both authors contributed to the article and approved the submitted version.

## Conflict of Interest

The authors declare that the research was conducted in the absence of any commercial or financial relationships that could be construed as a potential conflict of interest.

## References

[1] FAO/WUR. Expert consultation meeting: assessing the potential of insects as food and feed in assuring food security. In: Vantomme P, Mertens E, van Huis A, Klunder H, editors. Summary Report. Rome:FAO (2012). p. 23–5. Available online at: http://www.fao.org/docrep/015/an233e/an233e00.pdf (accessed January 30, 2021).

[2] Alexander P Brown C Arneth A Dias C Finnigan J Moran D. Could consumption of insects, cultured meat or imitation meat reduce global agricultural land use? Glob Food Sec. (2017) 15:22–32. 10.1016/j.gfs.2017.04.001

[3] Alexander P Rounsevell MDA Dislich C Dodson JR Engström K Moran D. Drivers for global agricultural land use change: the nexus of diet, population, yield and bioenergy. Glob Environ. Change. (2015) 35:138–47. 10.1016/j.gloenvcha.2015.08.011

[4] Smith P Gregory PJ. Climate change and sustainable food production. Proc Nutr Soc. (2013) 72:21–8. 10.1017/S0029665112002832 23146244

[5] Bustamante M Robledo-Abad C Harper R Mbow C Ravindranat NH Sperling F. Co-benefits, trade-offs, barriers and policies for greenhouse gas mitigation in the agriculture, forestry and other land use (AFOLU) sector. Glob Change Biol. (2014) 20:3270–90. 10.1111/gcb.12591 24700759

[6] Humpenöder F Popp A Dietrich JP Klein D Lotze-Campen H Bonsch M. Investigating afforestation and bioenergy CCS as climate change mitigation strategies. Environ Res Lett. (2014) 9: 064029. 10.1088/1748-9326/9/6/064029

[7] Van Huis A Van Itterbeeck J Klunder H Mertens E Halloran A Muir G. Edible Insects: Future Prospects for Food and Feed Security. Rome: FAO (2013).

[8] de Carvalho NM Madureira AR Pintado ME. The potential of insects as food sources–a review. Crit Rev Food Sci Nutr. (2020) 60:3642–52. 10.1080/10408398.2019.1703170 31868531

[9] Naseem R Majeed W Rana N Koch EB d A Naseem MR. Entomophagy: an innovative nutritional and economic navigational tool in race of food security. Int J Trop Insect Sci. (2020) 1–11. 10.1007/s42690-020-00284-8 Available online at: https://link.springer.com/article/10.1007/s42690-020-00284-8

[10] Doi H Gałecki R Mulia RN. The merits of entomophagy in the post COVID-19 world. Trends Food Sci Technol. (2021) 10:849–54. 10.1016/j.tifs.2021.01.067 33564209 PMC7862027

[11] Oonincx DG de BoerIJ. Environmental impact of the production of mealworms as a protein source for humans–A life cycle assessment. PLoS ONE. (2012) 7:e51145. 10.1371/journal.pone.0051145 23284661 PMC3526541

[12] Sánchez-Muros MJ Barroso FG Manzano-Agugliaro F. Insect meal as renewable source of food for animal feeding: a review. J Cleaner Prod. (2014) 65:16–27. 10.1016/j.jclepro.2013.11.068

[13] Berggren Å Jansson A Low M. Approaching ecological sustainability in the emerging insects-as-food industry. Trends Ecol Evol. (2019) 34:132–8. 10.1016/j.tree.2018.11.005 30655013

[14] Collavo A Glew RH Huang YS Chuang LT Bosse R Paoletti MG. House cricket small-scale farming. Paoletti MG, editor. In: Ecological Implications of Minilivestock: Potential of Insects, Rodents, Frogs, and Snails. New Hampshire: Science Publishers (2005). p. 519–44. 10.1017/S0014479705333540

[15] Mulia RN Doi H. Global simulation of insect meat production under climate change. Front. Sustain Food Syst. (2019) 3:91. 10.3389/fsufs.2019.00091

[16] Veldkamp T Van Duinkerken G van Huis A Lakemond CMM Ottevanger E Bosch G. Insects as a Sustainable Feed Ingredient in Pig and Poultry Diets: A Feasibility Study. Lelystad: Wageningen UR Livestock Research (Report/Wageningen UR Livestock Research 638) (2012).

[17] Gianotten N Soetemans L Bastiaens L. Agri-food side-stream inclusions in the diet of alphitobius diaperinus part 1: impact on larvae growth performance parameters. Insects. (2020) 11:79. 10.3390/insects11020079 31979388 PMC7074158

[18] Bodirsky BL Rolinski S Biewald A Weindl I Popp A Lotze-Campen H. Global food demand scenarios for the 21st Century. PLoS ONE. (2015) 10:e0139201. 10.1371/journal.pone.0139201 26536124 PMC4633131

[19] Cirera X Masset E. Income distribution trends and future food demand. Philos Trans R Soc Lond B. (2010) 365:2821–34. 10.1098/rstb.2010.0164 20713387 PMC2935126

[20] Eitelberg DA van Vliet JV Verburg PH. A review of global potentially available cropland estimates and their consequences for model-based assessments. Glob Change Biol. (2015) 21:1236–48. 10.1111/gcb.12733 25205590

[21] R Core Team. R.: A Language and Environment for Statistical Computing. (2020). Vienna: R Foundation for Statistical Computing. Available online at: https://www.R-project.org/ (accessed January 30, 2021).

[22] Raheem D Raposo A Oluwole OB Nieuwland M Saraiva A Carrascosa C. Entomophagy: nutritional, ecological, safety and legislation aspects. Food Res Int. (2019) 126:108672. 10.1016/j.foodres.2019.108672 31732082

[23] Bang A Courchamp F. Industrial rearing of edible insects could be a major source of new biological invasions. Ecol Lett. (2020) 24:393–397. 10.22541/au.160044142.21990828 Available online at: https://onlinelibrary.wiley.com/doi/epdf/10.1111/ele.13646 33226184

